# Naringin protects human nucleus pulposus cells against TNF-*α*-induced inflammation, oxidative stress, and loss of cellular homeostasis by enhancing autophagic flux via AMPK/SIRT1 activation

**DOI:** 10.1155/2022/7655142

**Published:** 2022-02-27

**Authors:** Renchang Chen, Shang Gao, Huapeng Guan, Xin Zhang, Yuliang Gao, Youxiang Su, Yun Song, Yuehua Jiang, Nianhu Li

**Affiliations:** ^1^Shandong University of Traditional Chinese Medicine, Jinan 250355, China; ^2^Spinal Department of Orthopedics, The Affiliated Hospital of Shandong University of Traditional Chinese Medicine, Jinan 250011, China; ^3^Central Laboratory of Affiliated Hospital of Shandong University of Traditional Chinese Medicine, Jinan 250014, China

## Abstract

Activation of the proinflammatory-associated cytokine, tumor necrosis factor*-α* (TNF*-α*), in nucleus pulposus (NP) cells is essential for the pathogenesis of intervertebral disc degeneration (IDD). Restoring autophagic flux has been shown to effectively protect against IDD and is a potential target for treatment. The goal of this study was to explore particular autophagic signalings responsible for the protective effects of naringin, a known autophagy activator, on human NP cells. The results showed that significantly increased autophagic flux was observed in NP cells treated with naringin, with pronounced decreases in the inflammatory response and oxidative stress, which rescued the disturbed cellular homeostasis induced by TNF*-α* activation. Autophagic flux inhibition was detectable in NP cells cotreated with 3-methyladenine (3-MA, an autophagy inhibitor), partially offsetting naringin-induced beneficial effects. Naringin promoted the expressions of autophagy-associated markers via SIRT1 (silent information regulator-1) activation by AMPK (AMP-activated protein kinase) phosphorylation. Either AMPK inhibition by BML-275 or SIRT1 silencing partially counteracted naringin-induced autophagic flux enhancement. These findings indicate that naringin boosts autophagic flux through SIRT1 upregulation via AMPK activation, thus protecting NP cells against inflammatory response, oxidative stress, and impaired cellular homeostasis. Naringin can be a promising inducer of restoration autophagic flux restoration for IDD.

## 1. Introduction

Intervertebral disc (IVD) is a composite tissue, composed of the inner nucleus pulposus (NP) and the outer annulus fibrosus [1]. NP cells are the predominant cell type within NP and control the metabolism in the extracellular matrix (ECM) of gelatinous NP by producing type II collagen (collagen II) and aggrecan, which are vital in resistance to compressive axial forces and pressure on the spine [1, 2].

Intervertebral disc degeneration (IDD) is a multi-factorial musculoskeletal disorder characterized by metabolic and structural changes that incrementally lead to the loss of IVD structural integrity and functions [3, 4]. It is estimated that IDD can be identified in roughly 40% of low back pain (LBP) patients [4] and is considered a significant contributor to the disease [5]. Moreover, the years lived with disability caused by LBP have been raised to 54% in 2015 from 1990, primarily attributed to population increase and ageing [6]. With this trend in demographic characteristics, the incidence of IDD will be likely to increase exponentially [7]. New treatment strategies for IDD have been studied for many years with limited achievements, due to unclear mechanisms for pathogenesis, which pose a challenge to effective precaution.

IDD has been proven to be involved in aberrant NP cell behavior stimulated by inflammatory cytokines [8–10]. Among them, TNF*-α* is most critical as it triggers a proinflammatory cascade [11], leading to mitochondrial dysfunction, elevated reactive oxygen species (ROS) in a dynamic equilibrium, and the resultant enhancement of oxidative stress in NP cells [12]. Persistent oxidative stress stimulates excessive catabolic enzyme accumulation [13], ECM degradation, cell phenotypic diversification, and even cell apoptosis [14]. All these constitute a complicated ROS-sensitive signaling network.

Macroautophagy is an evolutionarily conserved stress-responsive process that disposes unusual or damaged cytoplasmic organelles (e.g., damaged mitochondria) released from double-membrane vesicles (autophagosomes) and transports them to lysosomes for degradation, producing nutrients that contribute energy for cell metabolism [15]. Autophagic flux is a complete, dynamic process of autophagosome formation and fusion with lysosomes, cargo degradation, and the release of degradation products [11] in response to undernutrition, growth factor deficiency, hypoxia, infection, and other circumstances threatening cell survival. The overexpression of autophagy-associated markers is found to fuel cell apoptosis [16], which are critical targets for cell rescue. Some downstream components of the AMP-activated protein kinase (AMPK) signaling have been proven to have various roles, including an autophagic effect, in mitochondrial homeostasis [17, 18]. Among others, silent information regulator-1 (SIRT1) activated by AMPK is involved in the regulation of physiological and pathological cell processes like mitochondrial function [19], inflammation, cell apoptosis [20], and autophagy [21]. Therefore, the AMPK/SIRT1 signaling may serve as a potential target for IDD treatment.

Naringin, a major flavonoid identified in grapefruit, has been found to possess a wide variety of pharmacological properties including antioxidative, anti-inflammatory, and antiapoptotic effects [22–24], which are associated with restoration of the autophagic flux. In this study, we aimed to explore autophagic signalings responsible for those benefits of naringin's effects on TNF*-α*-treated NP cells—an *in-vitro* IDD model.

## 2. Materials and Methods

### 2.1. Ethics Statement

All procedures followed were performed in strict accordance with the ethical standards of the Ethics Committee of Shandong University of Traditional Chinese Medicine Affiliated Hospital on Human Experimentation and with the Helsinki Declaration (No. KY007). Informed consent was obtained from all participants included in the study.

### 2.2. Disc Harvest and NP Cell Culture

As previously mentioned [24], human NP cells were isolated and cultured. Briefly, human NP samples were acquired from 12 patients (6 males and 6 females; age range: 25–70 years) with a diagnosis of idiopathic scoliosis who were undergoing surgery. NP tissues were washed three times with D-Hank's (H1046, Solarbio, Peking, China) and cut into 1 mm^3^ fragments. Then, the tissues were treated with Trypsin-EDTA (0.25%; 59428C, Gibco, California, USA) for 30 min and type II collagenase (0.2%; C8150-100, Solarbio) for 4 h in a 37°C constant temperature water bath. Next, the filtered cells were transferred to DMEM/F12 containing 10% fetal bovine serum (FBS; S711-001S, Lonsera, Shanghai, China) and 1% penicillin-streptomycin (TMS-AB2, Gibco) in an incubator maintained at 37°C under a 5% CO_2_ atmosphere. The complete medium was changed every 2 to 3 days. After being identified using fluorescently labeled antibody for NP cell marker CD24 (11-0247-42, Thermo Fisher Scientific, Massachusetts, USA), the second passage cells were used for all experiments.

### 2.3. Cell Treatment

Recombinant human TNF-*α* (96-300-01A, Peprotech, Rocky Hill, NJ, USA) was prepared in sterile distilled H_2_O with 0.1% bovine serum albumin (BSA) and diluted in DMEM/F12 before use. Naringin (71162, Sigma-Aldrich, St. Louis, MO, USA) was directly prepared from DMSO (ST038, Beyotime Biotechnology, Shanghai, China) and diluted in DMEM/F12 to keep with 0.1% DMSO before use. The NPCs were exposed to the following treatments: (1) control group: DMEM/F12; (2) DMSO group: DMEM/F12 containing 0.1% DMSO; (3) TNF-*α* group: DMEM/F12 with TNF-*α* (50 ng/mL) containing 0.1% DMSO; (4) naringin group: DMEM/F12 with naringin (10 *μ*g/mL) containing 0.1% DMSO; (5) TNF-*α*+naringin group: DMEM/F12 with TNF-*α* (50 ng/mL) and naringin (10 *μ*g/mL) containing 0.1% DMSO; (6) TNF-*α*+naringin+3-MA (autophagy inhibitor; S2767, Selleck, Texas, USA) group: DMEM/F12 with TNF-*α* (50 ng/mL), naringin (10 *μ*g/mL), and 3-MA (10 *μ*M) containing 0.1% DMSO; (7) TNF-*α*+naringin+BML-275 (AMPK inhibitor; S7840, Selleck) group: DMEM/F12 with TNF-*α* (50 ng/mL), naringin (10 *μ*g/mL), and BML-275 (100 *μ*M) containing 0.1% DMSO; and (8) TNF-*α*+naringin+SIRT1-siRNA group: DMEM/F12 with TNF-*α* (50 ng/mL), naringin (10 *μ*g/mL), and SIRT1-siRNA containing 0.1% DMSO. Every group contains 0.1% DMSO except the control group.

NP cells were exposed to TNF-*α* (50 ng/mL) for 24 h to induce oxidative stress or were pretreated with naringin (10 *μ*g/mL) for 24 h alone or in combination with 3-MA (10 *μ*M) or BML-275 (100 *μ*M) for 2 h prior to treatment with TNF-*α* (50 ng/mL) for a further 24 h. For TNF-*α*+naringin+SIRT1-siRNA group, NP cells were transfected for 24 h with 100 pM siRNA against SIRT1 using Lipofectamine ® 2000 or were pretreated with naringin (10 *μ*g/mL) for 24 h alone.

### 2.4. Cell Viability Assay

The viability of the NP cells was evaluated by a Cell Counting Kit-8 (CCK-8; CK04, Dojindo Laboratories, Tokyo, Japan) assay in accordance with the manufacturer's instructions. Briefly, 8 × 10^4^ cells/well were seeded in 96-well plates and treated with naringin and TNF*-α* after cell adhesion as described above. At the indicated time, all NP cells collected were washed with phosphate-buffered saline (PBS, Beyotime Biotechnology). Then, 100 *μ*L of DMEM/F12 containing 10 *μ*L of CCK-8 solution was added to the cells in each well followed by incubation for 2 h at 37°C. After that, the OD was measured at 450 nm using an automicroplate reader (Bio-Tek ELx808TM, Vermont, USA).

### 2.5. ROS Assay

After various treatments, ROS production in NP cells was detected by a ROS assay kit (S0033S, Beyotime Biotechnology) according to the manufacturer's protocol. Cells were mixed with 1 mL of diluted DCFH-DA at 37°C for 20 min. The residual DCFH-DA liquid was removed after the cells were washed with PBS three times. The expression of green fluorescence was observed under a fluorescence microscope (Olympus IX71, Tokyo, Japan).

### 2.6. Superoxide Dismutase (SOD) Assay

SOD activity was measured using the SOD Typed Colorimetric Assay Kit (E-BC-K022-M, Elabscience Biotechnology, Wuhan, China). First, cell culture medium was collected and incubated with different test compounds according to the manufacturer's protocol. Next, an automicroplate reader (Bio-Tek ELx808TM) was used to read the absorbance of each sample at 550 nm. The absorbance value representing the intracellular SOD concentration of the tested NP cells.

### 2.7. ATP Assay

The intracellular ATP level was measured by the colorimetric luminescence method using an ATP assay kit (E-BC-K157-M, Elabscience Biotechnology). Treated and untreated cell samples were prepared. Then, ATP was extracted from NP cells with ATP extraction reagent to determine at 636 nm using an automicroplate reader (Bio-Tek ELx808TM). The relative luminescence value represented the intracellular ATP concentration of the tested NP cells.

### 2.8. Mitochondrial Membrane Potential (*ΔΨ*m) Assay

*ΔΨ*m changes in human NP cells were assessed using an JC-1 Assay Kit (C2003S, Beyotime Biotechnology). Briefly, collected cells after indicated treatments were incubated with an equal volume of JC-1 staining solution (5 *μ*g/mL) at 37°C for 20 min and rinsed twice with JC-1 staining buffer (1x). *ΔΨ*m changes were monitored by determining the relative amounts of dual emissions from mitochondrial JC-1 monomers or aggregates using a flow cytometry (BD Biosciences, New Jersey, USA).

### 2.9. MitoTracker Detection

MitoTracker staining (C1049B, Beyotime Biotechnology) was performed to visualize bioactive mitochondria and mitochondrial membrane potential changes in the human NP cells. NP cells were induced in 24-well plates according to the experimental protocol. The next step, wash with washing buffer 3 times. Typically, 188 *μ*L Annexin-V-FITC combined with liquid and 5 *μ*L Annexin-V-FITC dye liquid were added in turn to NP cells, and subsequently, 2 *μ*L MitoTracker red CMXRos was poured into the mixed system. All cells were incubated for 30 min at 25°C in the dark by using aluminum foil. The expression of MitoTracker Red and green CMXRos fluorescence was detected under a fluorescence microscope (Olympus IX71). Those living cells were marked as red fluorescence that maintain mitochondrial membrane potential while others were marked as green fluorescence that have undergone apoptosis or necrosis.

### 2.10. Apoptosis Detection

NP cells from each treatment group were labeled by double staining using an Annexin-V/PI Apoptosis Detection Kit (C1062S, Beyotime Biotechnology). According to the experimental protocol, 6 × 10^6^ suspended cells were collected for each cell sample. Five hundred microliters of diluted 1×Annexin-V binding buffer was added to the NP cells. Then, 5 *μ*L of Annexin-V-FITC staining solution and 5 *μ*L of nuclear DNA staining solution were added to the cell suspension. After mixing by gently vortexing, the cells were incubated at room temperature in the dark for 15~20 min. These cells were immediately detected with flow cytometry (BD Biosciences) after the reaction.

### 2.11. pH Measurements

Cultured NP cells (5 × 10^5^/mL) were seeded in 6-well plates. Upon request, NP cells were treated with drugs for 24 h until the cells reached 80% confluence. And NP cell culture medium from each treatment group was harvested, and extracellular pH (pHe) values were assessed by pH-indicator paper (1.09557, Sigma-Aldrich) and pH meter (SX5150, Sanxin, Shanghai, China) and compared with those of the control.

### 2.12. Alcian Blue Staining

The glycosaminoglycan production of the NP cells was measured using a Alcian Blue Stain Kit (G1560, Solarbio) according to the manufacturer's instructions. Briefly, NP cells were first fixed with 4% paraformaldehyde for 40 min. After 5 min of nuclear fast red staining, alcian acidification solution (alcian acidification solution: distilled water = 1 : 2) was added to the cells to soak for 5 min. Then, Alcian staining solution was added to the dye for 30 min. Finally, the cells on cover slips were observed and photographed under an inverted microscope (Leica DMi1, Wetzlar, Germany).

### 2.13. Collagen II Immunocytochemical Staining

All collected NP cells were cultured in 6-well plates with coverslips at 1 × 10^5^ cells/mL, treated as mentioned above and fixed with fresh 4% paraformaldehyde for 40 min. Then, the NP cells were incubated with 0.3% Triton X-100 (ST795, Beyotime Biotechnology) for 20 min. The samples were blocked with 5% bovine serum albumin for 60 min. The cells were then incubated with the primary antibody in proportion against collagen II (GB11022, 1 : 200, Servicebio, Wuhan, China) at 4°C overnight. After incubation with the primary antibody, a secondary antibody was diluted in proportion (GB23303, 1 : 1000, Servicebio) and added to the cells for 30 min. The cells were incubated with DAB reagent for 10 min followed by hematoxylin staining for 20 s at room temperature. After washing with PBS, the stained NP cells were examined and imaged using an inverted microscope (Leica DMi1).

### 2.14. Transmission Electron Microscopy (TEM)

In order to observe the ultrastructure of the autophagosomes and autolysosomes, second passage NP cells were fixed in 2.5% glutaraldehyde solution for 12 h after treatment and then fixed again with 1% osmium acid. Following gradient dehydration with a series of ethanol and acetone solutions, the cells were embedded in epoxy resin, and the selected areas were cut into 70 nm ultrathin sections. The cells were then stained with lead citrate and uranium acetate. Subsequently, all of the ultrathin sections were observed under a TEM (JEM-ARM200F, Tokyo, Japan) to calculate the number of autophagosomes.

### 2.15. pAV-CMV-TagRFP-SEP-LC3 Assay

To estimate the efficiency of autophagic flux, the NP cells were dealt with pAV-CMV-TagRFP-SEP-LC3 recombinant adenovirus vectors (S2231, Sunbio Medical Biotech Co., Ltd., Shanghai, China). First, the cells were inoculated in 24 aperture plates with a density of 1 × 10^5^ cells/mL. Next, the pAV-CMV-TagRFP-SEP-LC3 recombinant adenovirus titer of the original solution was diluted to 1 × 10^8^ PFU/mL with PBS. When the MOI = 100, the transfection efficiency was the best. A precalculated adenovirus volume was added to the cells for 24 h. On the following day, the existing culture medium was replaced and the designated drug was added. After treatment, the cells were observed and measured by the laser-scanning confocal microscope (Dragonfly200, Andor, UK). As discussed earlier [25], autophagosomes appear as yellow spots, but autolysosomes appear as red spots. With increase of the autophagic flux, both yellow and red spots increase correspondingly. But while autophagic flux is blocked, yellow spots are increased but red spots are kept also.

### 2.16. TUNEL Staining

To examine the apoptosis of human NP cells in each indicated group, cells were stained with a One-Step TUNEL Apoptosis Assay Kit (C1088; Beyotime Biotechnology). Briefly, the cultured NP cells were prepared in a 6-wll plate. After treatment, these cells were fixed with freshly prepared 4% paraformaldehyde for 1 h, incubated with 0.1% Triton X-100 for 10 min, and washed with PBS three times in every step. According to the manufacturer's instructions, cells were stained with an in situ cell death detection kit. Apoptotic changes were measured under a fluorescence microscope (Olympus IX71).

### 2.17. Immunofluorescence Staining

Expression of matrix macromolecules (collagen II; ab283694, Abcam, Cambridge, UK) and catabolism enzymes (MMP-3; ab137659, Abcam) and autophagy-related markers (LC3; ab128025, Abcam) was analyzed by immunofluorescence. NP cells were fixed to glass coverslips with 4% paraformaldehyde (P0099, Beyotime Biotechnology) for 40 min and then permeabilized with 0.1% Triton X 100 (ST797, Beyotime Biotechnology) for 20 min. After blocking with 0.5% BSA for 30 min, the NP cells were incubated with primary antibodies against collagen II (1 : 200) and MMP-3 (1 : 200) at 4°C overnight. The next day, the NP cells were incubated with the appropriate secondary antibody at room temperature in the dark for 60 min. After washing with PBS, the cells were incubated with DAPI at room temperature for 3 min. Fluorescence images were acquired by a fluorescence microscope (Olympus IX71).

### 2.18. siRNA Transfection

Before siRNA transfection, 5 × 10^4^ cells/mL were seeded in a 6-well plate. The experiment was carried out until the cells reached 60% confluence. Human SIRT1 siRNA and the negative control siRNA (NC-siRNA) were designed and synthesized by GenePharma (Shanghai, China). A hundred pM of siRNA was mixed gently with 250 *μ*L of Opti-MEM medium. Four microliters of diluted Lipofectamine® 2000 (11668030, Invitrogen, California, USA) reagent was gently mixed with 196 *μ*L of Opti-MEM medium at room temperature for 5 min. The diluted siRNA and RNAi-Mate reagent was gently mixed with the siRNA/Lipofectamine® 2000 complex at room temperature for 20 min. The complex was added to the well of the culture plate, and the cell culture plate was gently shaken back and forth. After the cells were incubated at 37°C in a humidified 5% CO_2_ atmosphere for 24 h, the other detection steps after transfection were carried out.

### 2.19. Quantitative Real-Time PCR (qRT-PCR)

The expression levels of MMP-3 and collagen II were detected by qRT-PCR. After various treatments, the total mRNA of NP cells was extracted by the column method (AC0203, Sparkjade). GAPDH was used to normalize gene expression, and complementary DNA (cDNA) was synthesized at 50°C for 30 min (AG0304, Sparkjade). The PCR conditions were as follows: denaturing at 94°C 3 times for 1 min, annealing at 60°C for 20 s, extension at 72°C for 30 s, and finally amplification once. Next, SYBR Green PCR master mix (AH0104-C, Sparkjade) on a Roche LightCycler®480 instrument (Roche, Basel, Switzerland) was further used for qRT-PCR. The synthetic primer sequences were as follows: human GAPDH (F) 5′-GAAGGTGAAGGTCGGAGTC-3′, (R) 5′GAAGATGGTGATGGGATTTC-3′; MMP3 (F) 5′-GGGTCTCTTTCACTCAGCCAACAC-3′, (R) 5′ACAGGCGGAACCGAGTCAGG-3′; and collagen II (F) 5′-TGAAGACCCAGACTGCCTCAA-3′, (R) 5′-CGAGGTCAGCTGGGCAGAT-3′. The relative expression of RNA was calculated by the 2^-*ΔΔ*CT^ method.

### 2.20. Western Blot Assay

Total protein was extracted from NPCs using the RIPA buffer (P0013K, Beyotime Biotechnology) with 1 mmol/L PMSF. The protein concentration was measured using a BCA protein assay kit (P0012, Beyotime Biotechnology) and Skanit software 3.1 for Multiskan Go (Thermo Fisher Scientific). The protein extracted from each sample was electrophoretically separated on a 5-20% prefabricated polyacrylamide gel and transferred to a polyvinylidene fluoride (PVDF; BS-PVDF-45-22, Biosharp, Peking, China) membrane. The membrane was blocked for 1 h with 5% nonfat milk and incubated with primary antibodies overnight at 4°C: COX-2 (1 : 1000; ab179800, Abcam), C-caspase-3 (1 : 500; ab32351, Abcam), Bax (1 : 1000; ab32503, Abcam), Bcl-2 (1 : 1000; ab32124, Abcam), LC3 (1 : 1000; ab128025, Abcam), Beclin-1 (1 : 300; ab217179, Abcam), P62 (1 : 1000; ab182579, Abcam), MMP-3 (1 : 500; ab137659, Abcam), ADAMTS-4 (1 : 500; ab84792, Abcam), collagen II (1 : 5000; ab283694, Abcam), aggrecan (1 : 1000; ab36861, Abcam), AMPK (1 : 1000; ab23875, Abcam), p-AMPK (1 : 1000; ab23875, Abcam), and SIRT1 (1 : 1000; ab7343, Abcam). Then, the membrane was incubated with horseradish peroxidase- (HRP-) bound secondary antibody (1 : 5000; ab7090, Abcam) for 2 h at room temperature. The membranes were visualized using a Millipore ECL chemiluminescence kit (WBKLS0100, Merck) and exposed to a autoradiographic film. Finally, target protein expression normalized to GAPDH was analyzed using ImageJ software version 1.8.0 (National Institutes of Health, USA).

### 2.21. Statistical Analysis

Data are presented as the means ± standard deviation (SD). All statistical analyses were performed using GraphPad Prism version 8.0.1 (GraphPad Software Inc., California, USA). Intergroup differences were analyzed by one-way analysis of variance (ANOVA), and Tukey's test was used to compare the control group and the treatment group. A value of *p* < 0.05 was considered statistically significant. All experiments were performed in triplicate.

## 3. Results

### 3.1. Naringin Rescues Human NP Cells Treated with TNF-*α*

**Figure 1:**
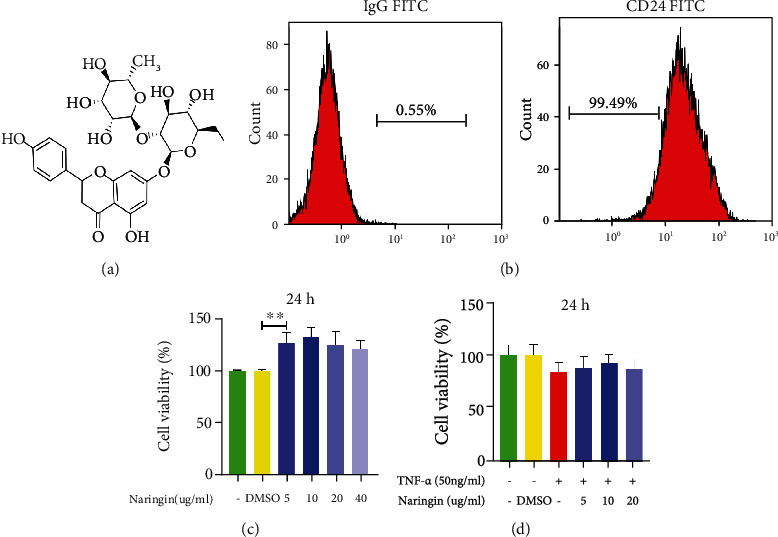
Effect of naringin on TNF-*α*-induced human NP cells. (a) Chemical structure of naringin. (b) Identification of human NP cell marker CD24 by flow cytometry. (c) CCK-8 results of human NP cells treated with different concentrations of naringin contain 0.1% DMSO for 24 h. (d) CCK-8 results of naringin pretreated human NP cells induced by TNF-*α* for 24 h. The data represent the means ± SD. Significant differences between groups are indicated as ^∗^*p* < 0.05, ^∗∗^*p* < 0.01, and ^∗∗∗^*p* < 0.001, *n* = 3.

The chemical structure of naringin is shown in Figure 1(a). Human NP cells from all 12 cases showed high CD24^+^ (99.49 ± 0.19%; range 99.3–99.68%) expression under normal conditions (Figure 1(b)). In the CCK-8 assay, no marked increase in naringin cytotoxicity (0, 0.1% DMSO, 5, 10, 20, and 40 *μ*g/mL of naringin) was detected in NP cells at 24 h (Figure 1(c)). 10 *μ*g/mL naringin pretreatment showed the strongest protective effects against TNF*-α* (50 ng/mL) (Figure 1(d)). These results determined that the optimal dose of naringin for NP cell rescue was 10 *μ*g/mL.

### 3.2. Naringin Attenuates TNF-*α*-Induced Inflammation in Human NP Cells

**Figure 2:**
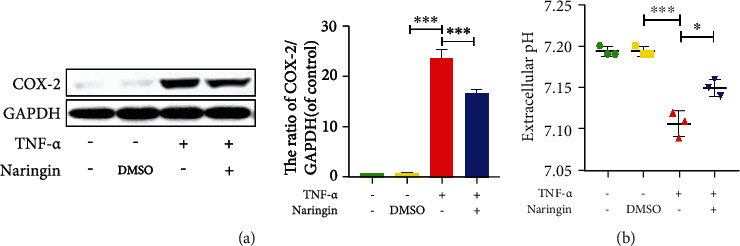
Effect of naringin on TNF-*α*-induced microinflammatory environment. (a) The level of COX-2 protein was measured by Western blotting. (b) Extracellular pH was detected by pH-indicator paper and pH meter. The data represent the means ± SD. Significant differences between groups are indicated as ^∗^*p* < 0.05, ^∗∗^*p* < 0.01, and ^∗∗∗^*p* < 0.001, *n* = 3.

TNF*-α*, a spark for high-grade inflammation, stimulates the release of inflammatory mediators and further leads to degenerative changes [26]. In this study, elevated COX-2 levels were detectable in NP cells treated for 24 h with TNF*-α*, which was significantly suppressed in cells pretreated with naringin (Figure 2(a)). These data support the reliability of the TNF*-α*-induced inflammation model *in vitro*.

An acidic microenvironment is often a result of local inflammation [27]. We detected extracellular pH (pHe) to explore the anti-inflammatory properties of naringin against TNF-*α*. A lower pHe was observed in TNF*-α*-treated cells versus intact and DMSO-treated controls. But naringin pretreatment markedly elevated the pHe (Figure 2(b)). Therefore, naringin significantly counteracts the adverse effects of TNF-*α* on NP cells, demonstrating a robust anti-inflammatory activity in the *in vitro* IDD model.

### 3.3. Naringin Inhibits TNF-*α*-Induced Oxidative Stress and Mitochondrial Dysfunction in Human NP Cells

**Figure 3:**
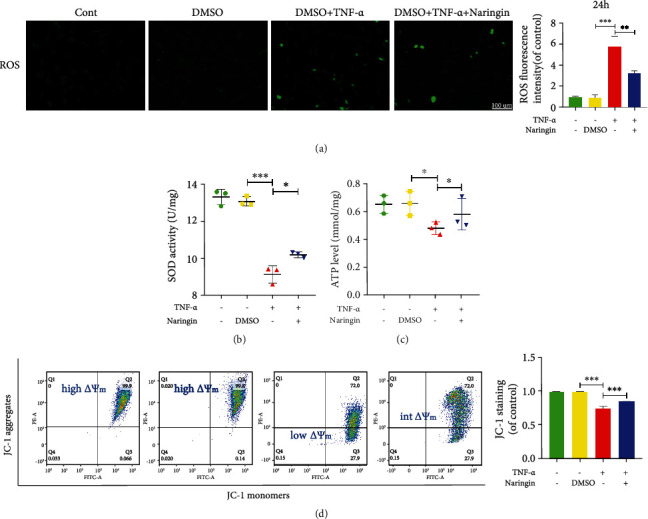
Effect of naringin on TNF-*α*-induced oxidative stress and mitochondrial dysfunction. (a) ROS fluorescence under fluorescence microscope (scale bar = 100 *μ*m). (b, c) Intracellular SOD activity and ATP levels in human NP cells. (d) Mitochondrial membrane potential (*ΔΨ*m) was detected by JC-1 staining and measured by flow cytometry. The data represent the means ± SD. Significant differences between groups are indicated as ^∗^*p* < 0.05, ^∗∗^*p* < 0.01, and ^∗∗∗^*p* < 0.001, *n* = 3.

Oxidative stress activation has been implicated in excessive ROS-induced mitochondrial dysfunction and the process of IDD [25]. So we assessed intracellular H_2_O_2_ and oxidative stress in mitochondria after TNF*-α* versus naringin treatment using a DCFH-DA fluorescent probe. Then, significantly enhanced ROS generation was observed in NP cells treated with TNF*-α* alone, which was partially counteracted by naringin (Figure 3(a)). Superoxide dismutase (SOD) is a key intracellular antioxidant from the mitochondrial matrix that catalyzes the conversion of ^·^O_2_^−^ into H_2_O_2_. The latter decompose to H_2_O and O_2_ [25, 28]. As shown in Figure 3(b), the SOD activity was significantly lower in TNF-*α*-treated NP cells versus intact and DMSO-treated controls. Naringin boosted the SOD activity compared to that achieved in cells treated with TNF-*α* alone. Diminished mitochondrial transmembrane potential (*ΔΨ*m) permeability and ATP synthesis is evidence of mitochondrial dysfunction. TNF-*α* treatment led to a loss of *ΔΨ*m and reduced ATP levels in human NP cells (Figures 3(d) and 3(c)). Naringin partially restored the deficiency in TNF-*α*-treated cells.

### 3.4. Naringin Suppresses TNF-*α*-Induced Apoptosis in Human NP Cells

**Figure 4:**
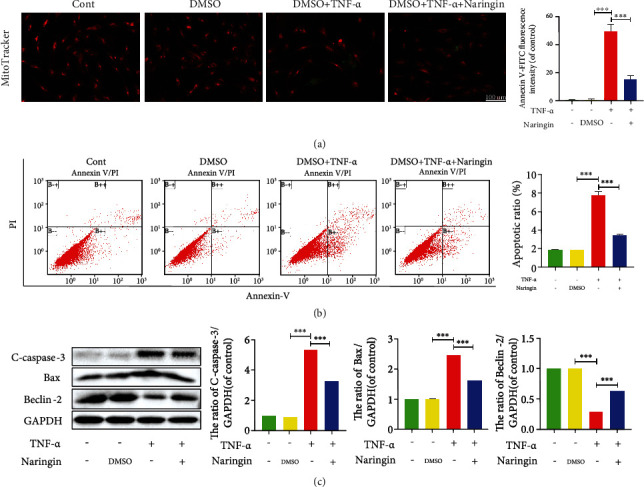
Naringin inhibits TNF-*α*-induced human NP cell apoptosis. (a) MitoTracker Red CMXRos fluorescence under fluorescence microscope (scale bar = 100 *μ*m). (b) Annexin-V/PI staining to detect the rate of apoptosis in human NP cells. (c) The protein levels of C-caspase-3, Bax, and Beclin-2 in cells were measured by Western blotting. The data represent the means ± SD. Significant differences between groups are indicated as ^∗^*p* < 0.05, ^∗∗^*p* < 0.01, and ^∗∗∗^*p* < 0.001, *n* = 3.

Several pore-forming members of the Bcl-2 protein family (e.g., Bax) mediate the permeability of the mitochondrial outer membrane [29]. We employed MitoTracker staining, which labels mitochondria of live cells, to assess NP cell apoptosis. As shown in Figure 4(a), MitoTracker red fluorescence (live staining) significantly decreased, and MitoTracker green fluorescence (dead staining) was pronouncedly enhanced after TNF-*α* treatment. An opposite finding was shown in cells pretreated with naringin compared to the TNF-*α* group, indicating that naringin potently restores mitochondrial function and NP cell activity. Unlike the intrinsic (mitochondrial) apoptotic pathway, TNF-*α* elevates extracellular Fas and FasL levels to activate the initiator caspases-3, followed by activation of the apoptotic signaling cascade in cells [30, 31]. Western blot assay revealed an apoptotic effect of TNF-*α* in NP cells, characterized by significantly elevated proapoptotic Bax and cleaved-caspase-3 (C-caspase-3) and suppressed antiapoptotic Bcl-2 expression (Figure 4(c)), which was also evidenced by the flow cytometry results (Figure 4(b)). Overall, TNF-*α* triggers NP cell apoptosis via both the mitochondrial pathway and the death receptor pathway. Naringin pretreatment can significantly eliminate the proapoptotic activity.

### 3.5. Naringin Ameliorates ECM Degradation in TNF-*α*-Treated Human NP Cells

**Figure 5:**
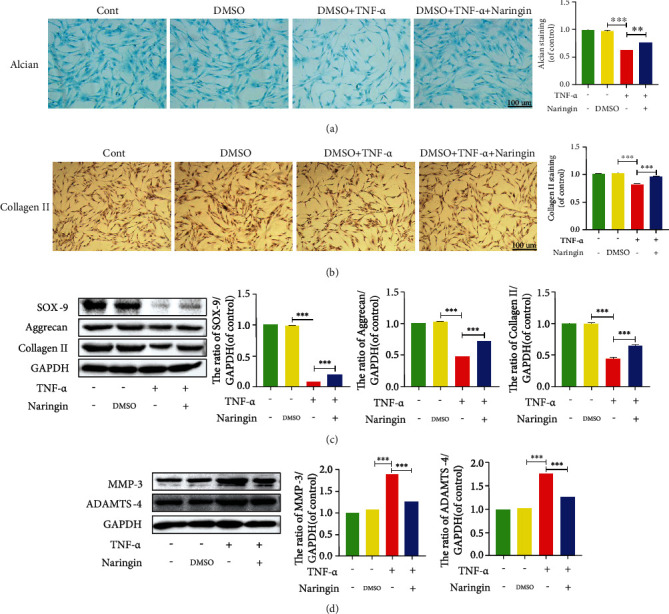
Naringin ameliorates ECM degradation in TNF-*α*-induced human NP cells. (a) Alcian blue staining in human NP cells under inverted microscope (scale bar: 100 *μ*m). (b) Collagen II staining in human NP cells under inverted microscope (scale bar: 100 *μ*m). (c, d) The protein levels of SOX-9, aggrecan, collagen II, MMP-3, and ADAMTS-4 were measured by Western blotting. The data represent the means ± SD. Significant differences between groups are indicated as ^∗^*p* < 0.05, ^∗∗^*p* < 0.01, and ^∗∗∗^*p* < 0.001, *n* = 3.

IDD is mostly associated with the disequilibrium of ECM synthesis and degradation primarily mediated by matrix metalloproteinase (MMP) enzymes and a disintegrin and metalloproteinase with thrombospondin motifs (ADAMTS). We utilized the Alcian blue staining and collagen II immunohistochemistry to assess changes in ECM components. The results showed that TNF-*α* stimulation pronouncedly suppressed the synthesis of aggrecan (one of the most important structural proteoglycans) and collagen II (Figures 5(a) and 5(b)). The expressions of collagen II biomarkers, aggrecan and SOX-9 (for cartilage synthesis), were markedly inhibited by TNF-*α*. However, MMP3 and ADAMTS-4 expressions were enhanced after TNF-*α* administration (Figures 5(c) and 5(d)), indicating TNF-*α*-induced ECM loss. These aberrant expressions of ECM components were dramatically reversed by naringin.

### 3.6. Naringin Upregulates Autophagic Flux in Human NP Cells via Autophagy Activation

**Figure 6:**
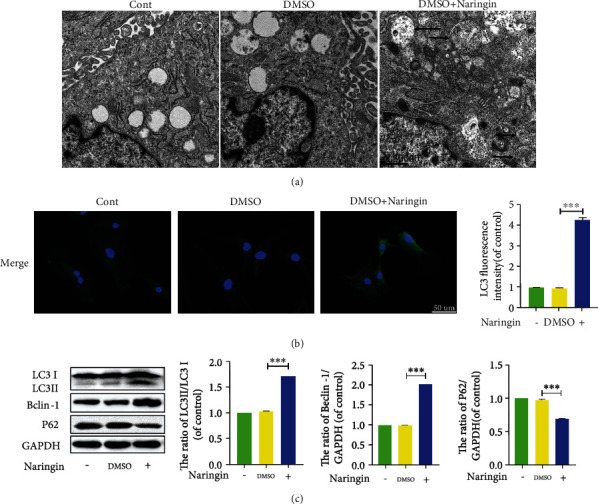
Regulation of autophagy in human NP cells cotreated with naringin. (a) Morphology of autophagosomes and autolysosomes was detected by transmission electron microscopy (×30000) in human NP cells (as indicated by black arrow). (b) The representative LC3 expression was detected by the immunofluorescence (scale bar: 50 *μ*m). (c) The protein levels of LC3, Beclin-1, and P62 in cells were measured by Western blotting. The data represent the means ± SD. Significant differences between groups are indicated as ^∗^*p* < 0.05, ^∗∗^*p* < 0.01, and ^∗∗∗^*p* < 0.001, *n* = 3.

We assessed the expressions of the autophagy-related markers LC3 and Beclin-1 using Western blot to explore whether single-use naringin could protect NP cells via inducing autophagy. P62 is a specific substrate degraded in autolysosomes. P62 protein levels were quantitated in this study to explore the impairment of autophagic flux. Naringin markedly increased LC3-II/I ratio and Beclin-1 expression and inhibited p62 expression in TNF-*α*-treated NP cells versus intact and DMSO-treated controls (Figure 6(c)), indicating ameliorated autophagy. This finding was also supported by the enhanced fluorescence intensity of LC3 (Figure 6(b)). The TEM observation showed that more autophagosomes were observed in cells pretreated with naringin (Figure 6(a)). All these results suggest that naringin triggers autophagy and promotes autophagic flux in NP cells.

### 3.7. The Protective Effects of Naringin Are Reversed by Autophagic Flux Inhibition

**Figure 7:**
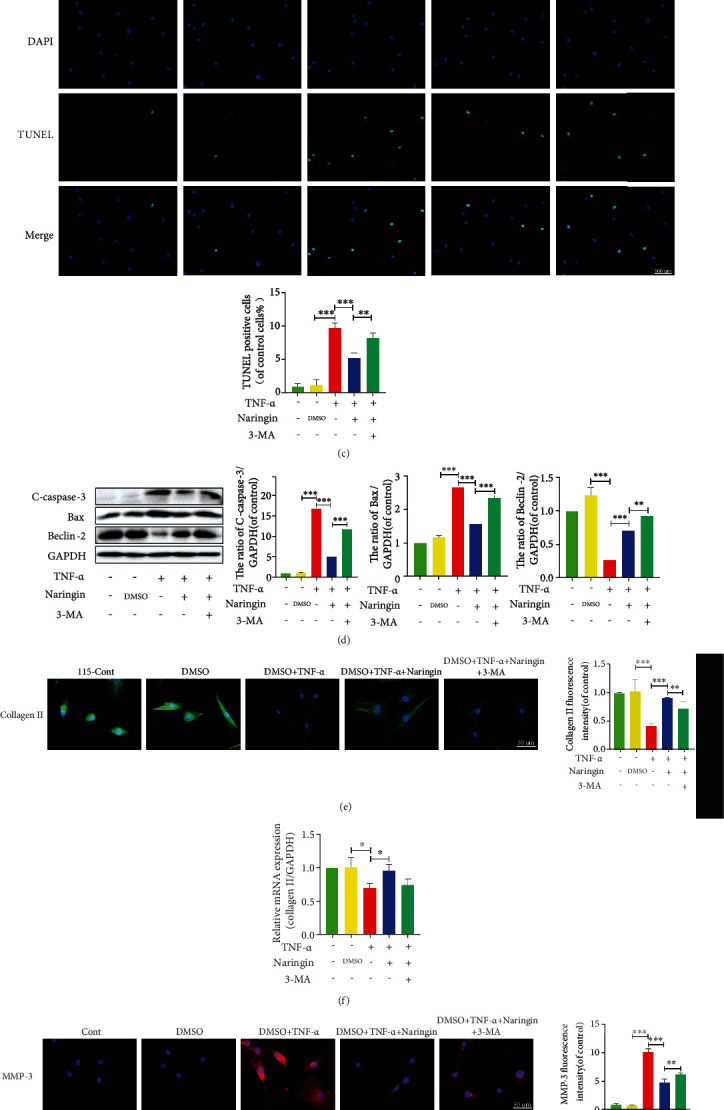
Inhibition of autophagy with 3-MA reverses the protective effect of naringin. (a) The protein levels of LC3, Beclin-1, and P62 were measured by Western blotting. (b) The variation of autophagic flux was detected by confocal microscope (yellow spots represent autophagosomes, red spots represent autolysosomes). (c) TUNEL staining results to detect the rate of apoptosis in human NP cells. () The protein expressions of C-caspase-3, Bax, and Beclin-2 were measured by Western blotting. (e, g) The distribution of Collagen II (green), MMP-3 (red), and DAPI (blue) were detected by the immunofluorescence (scale bar: 100 *μ*m). (f, h) The mRNA expressions of Collagen II were measure by qRT-PCR. (i) The protein expressions of Collagen II and MMP-3 were measured by Western blotting. The data represent the means ± SD. Significant differences between groups are indicated as ^∗^*p* < 0.05, ^∗∗^*p* < 0.01, and ^∗∗∗^*p* < 0.001, *n* = 3.

The autophagy inhibitor 3-MA was used to ascertain whether naringin's protection against TNF-*α*-induced IDD depended on autophagy stimulation. As shown in Figure 7(a), TNF-*α* partially increased LC3-II/LC3-I and Beclin-1 expressions and p62 expression. This may be related to NP cell defenses against pathological lesion via enhancing autophagy in the damaged areas through reutilizing their substances for energy. The pAV-CMV-TagRFP-SEP-LC3 assay revealed an increased number of yellow spots rather than red spots in TNF-*α*-treated cells, suggesting impaired autophagic flux (Figure 7(b)). Naringin induced an increment in autophagic flux under TNF-*α* exposure, but intriguingly, the protective effects of naringin were partially offset by 3-MA. So naringin may inhibit TNF-*α* via restoring autophagic flux for protection.

We next investigated whether these benefits from naringin-induced autophagy could ascribe to the efficient rebalancing of cell apoptosis and ECM degeneration in human NP cells treated with TNF-*α*. We detected apoptosis-related proteins and found that naringin repressed proapoptotic C-caspase-3 and Bax expressions that had elevated by TNF-*α* but increased Beclin-2 expression (Figure 7(d)). In the TUNEL staining, naringin suppressed the green fluorescence intensity once enhanced by TNF-*α* (Figure 7(c)), but this effect was counteracted by 3-MA. Also, 3-MA inhibited the expressions of collagen II biomarkers that had been improved and corrected the low MMP-3 expression in naringin-treated cells (Figures 7(e)–7(i)). These results indicate that 3-MA inhibits already ameliorated autophagic flux by naringin, canceling the benefits from naringin treatment (Figure 7).

### 3.8. Naringin-Mediated Autophagy Is Activated by the AMPK/SIRT1 Signaling in Human NP Cells

**Figure 8:**
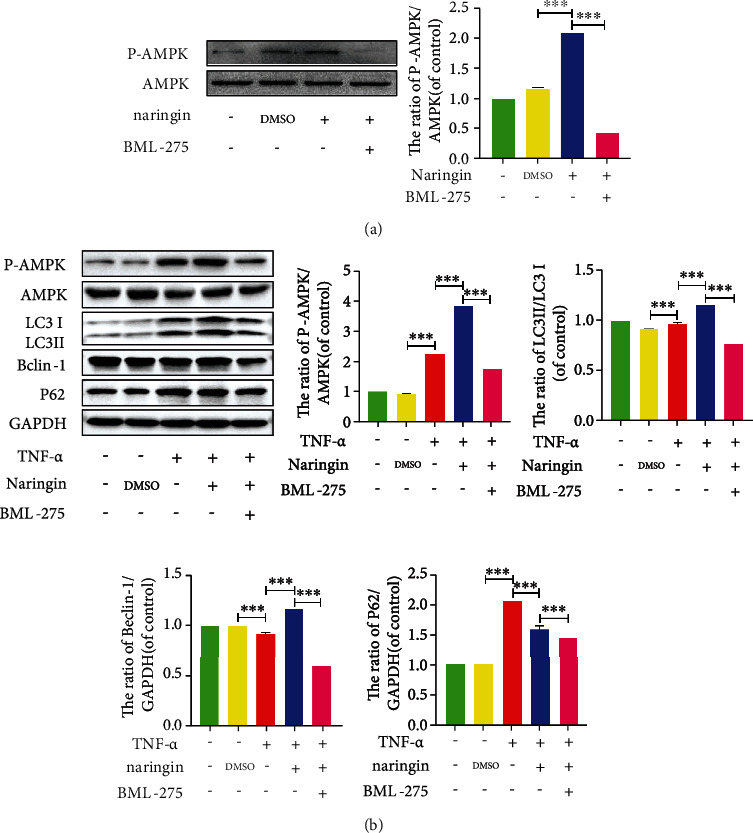
AMPK signaling is involved in naringin-triggered activation of autophagy in human NP cells. (a) The protein expressions of p-AMPK/AMPK were measured by Western blotting. (b) The protein levels of LC3, Beclin-1, and P62 were measured by Western blotting. The data represent the means ± SD. Significant differences between groups are indicated as ^∗^*p* < 0.05, ^∗∗^*p* < 0.01, and ^∗∗∗^p < 0.001, *n* = 3.

**Figure 9:**
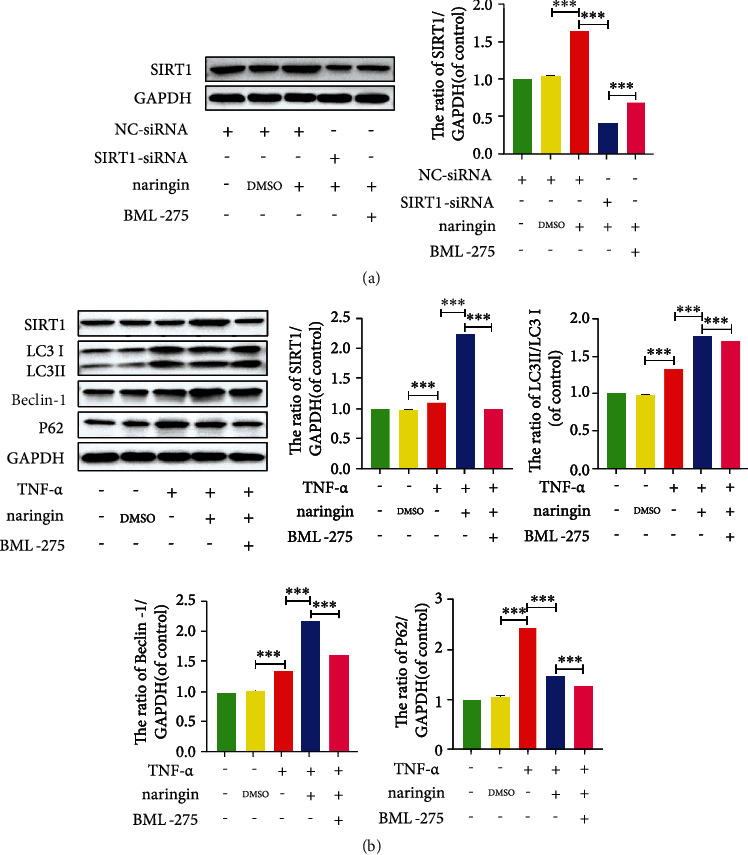
SIRT1 signaling is involved in naringin-triggered activation of autophagy in human NP cells. (a) The protein expressions of SIRT1 were measured by Western blotting. (b) The protein levels of LC3, Beclin-1, and P62 were measured by Western blotting. The data represent the means ± SD. Significant differences between groups are indicated as ^∗^*p* < 0.05, ^∗∗^*p* < 0.01, and ^∗∗∗^*p* < 0.001, *n* = 3.

**Figure 10:**
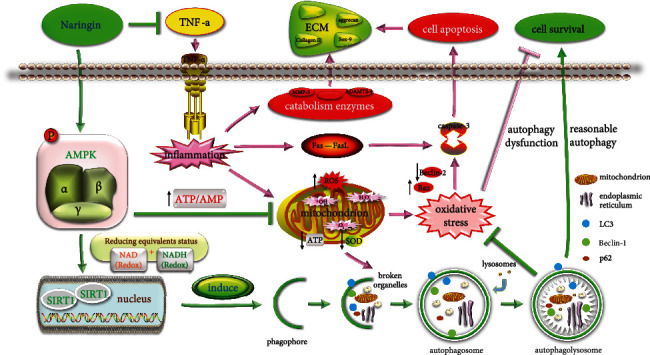
TNF-*α* is the key factor in IDD, which leads to human NP cell apoptosis in two ways: (1) TNF-*α* triggers inflammation and causes further oxidative stress in mitochondria, which gives rise to disordered metabolism and NP cell apoptosis. (2) TNF-*α* induces NP cell apoptosis through the death receptor pathway. The accumulation of apoptotic NP cells breaks the dynamic balance between the ECM destruction and synthesis via a malignant positive feedback loop, resulting in IDD and eventually LBP. Naringin, an autophagy inducer, regulates autophagy levels by activating AMPK and upregulating SIRT1 expression. This increase in autophagic flux inhibits the inflammatory cascade and ROS induced by TNF-*α* and phagocytizes useless organelles to provide more energy for NP cells. All of these events improve mitochondrial energy synthesis, decrease the expression of catabolic enzymes, and attenuate apoptosis of cells to effectively promote the formation of ECM to delay the process of IDD.

Since the AMPK/SIRT1 signaling has been shown to have the greatest therapeutic potential for IDD, we explored whether it could be responsible for naringin-induced autophagy in TNF-*α*-treated NP cells. The phosphor AMPK (p-AMPK) expression significantly increased in cells pretreated with naringin, whose p-AMPK/AMPK ratio could be suppressed by the AMPK inhibitor BML-275 (Figure 8(a)). Naringin elevated LC3 and Beclin-1 expressions, together with a lower p62 level and a rising p-AMPK/AMPK ratio in NP cells. However, the enhanced autophagy activity by naringin was further depressed after the addition of BML-275 (Figure 8(b)). As for the potential roles of SIRT1, the most significant downstream component of AMPK, we knocked down the SIRT1 gene by siRNA transfection. SIRT1 expression significantly decreased in negative controls and transfected cells pretreated with naringin compared to high SIRT1 expression in untransfected cells pretreated with naringin (Figure 9(a)). The high SIRT1 expression in naringin-treated untransfected cells was dramatically inhibited by the AMPK inhibitor BML-275 (Figures 9(a) and 9(b)). These findings suggest that naringin may activate autophagy activity via AMPK/SIRT1 activation (Figure 10).

## 4. Discussion

IDD often occurs prior to other musculoskeletal symptoms. It is reported that an estimated 20% of teens have developed mild-degenerated discs [3]; IDD often increases with age, and this trend is particularly apparent in males. Besides, 10% of 50-year-olds and 60% of 70-year-olds are found to have severe IDD [32]. Currently, the underlying mechanisms and treatment scenarios for IDD remains rather limited, despite the continuing impacts of this disease on life quality. Identification of active ingredients against IDD progression is urgently needed [25]. Naringin is a natural anti-inflammatory compound comprised of various pharmacological activities capable of dealing with degenerative-related diseases [23, 33, 34]. In the current study, we explored the mechanisms responsible for naringin's protection against TNF-*α* in an *in vitro* IDD model and ascertained that naringin-induced autophagic flux increment suppresses inflammation response, oxidative stress, cell apoptosis, and ECM degradation induced by TNF-*α* via AMPK/SIRT1 activation in human NP cells.

It is widely accepted that very few mitochondria are present in NP cells due to a physiologically avascular and hypoxic niche therein. However, Madhu et al. reported abundant mitochondria in NP cells based on *in vivo* and *in vitro* experiments [35]. ROS production (including ^·^O_2_^−^, H_2_O_2_, and ^·^OH) is required in oxidative metabolism to produce ATP in mitochondria. Mitochondrial SOD converts ^·^O_2_^−^ into H_2_O_2_, and the latter is translated by catalase into harmless H_2_O to maintain the antioxidant/oxidative balance of cells [27]. Nevertheless, an extracellular concentration of H_2_O_2_ may increase 10 to 100 times in response to inflammation [36]. TNF-*α* has been considered the culprit cytokine that triggers an inflammatory cascade [4, 9] via promoting the secretion of inflammatory mediators such as cyclooxygenase-2 (COX-2) in IDD [25]. Thus, we established the IDD model *in vitro* using TNF-*α*. In the present study, TNF-*α* significantly increased COX-2 levels, enhancing inflammation in human NP cells. It also elevated ROS and ATP production, SOD activity, and mitochondrial membrane potential, hallmarks of extensive oxidative stress in cells. In addition, extracellular pH was slightly inhibited after TNF-*α* exposure, creating a dynamic acidic niche in response to inflammation. This can also be explained by disrupted Ca^2+^ homeostasis under oxidative stress [37]. All these were potently attenuated by naringin pretreatment.

Loss of biochemical homeostasis in NP cells leads to cell apoptosis, exacerbating IDD [3]. Two apoptotic pathways, the mitochondrial (intrinsic) and death receptor (extrinsic) pathways, have been identified. For the former, oxidative stress contributes to cell apoptosis, leading to the imbalance between proapoptotic Bax and antiapoptotic Bcl-2, further stimulating caspase-3 activation and thereby accelerating NP cell apoptosis [38]. For the latter, extracellular TNF-*α* activates the effector caspase-3 to unleash intracellular apoptosis signals via the death receptor-mediated pathway [29]. In the present study, our findings demonstrated that TNF-*α* aggravated cell apoptosis in NP cells via a combination of the intrinsic and extrinsic pathways, whereas this damaging effect was overturned by naringin.

Excessive apoptosis in NP cells directly breaks the dynamic balance between the ECM destruction and synthesis [39]. Normally, the two predominant ECM components, aggrecan (covalently attaching highly anionic glycosaminoglycans) and collagen II, are responsible for cellular homeostasis. Further, SOX-9 has been proven to have a positive correlation with the number of NP cells and the presence of ECM. These resident cells, though limited (approximately 5,000 cells/mm^3^), are responsible for producing ECM and maintaining the integrity of the IVD [32]. Furthermore, numerous biomarkers for matrix-degrading enzymes (e.g., MMPs and ADAMTSs) play a crucial role in ECM degradation [39]. We found that naringin suppressed MMP-3 and ADAMTS-4 expressions and retained the matrix components by promoting type II collagen contents and aggrecan and SOX-9 expressions in TNF-*α*-treated NP cells, demonstrating its strong effect of protecting against ECM degeneration.

Autophagy degrades damaged organelles and proteins for nutrient recycling. During autophagy, Beclin-1 participates in the nucleation of the autophagosomal membrane [40], and LC3-II is involved in double-membrane vesicle formation and the elongation of the autophagosomal membrane [41], which are considered the biomarkers for autophagy initiation. In autophagosomes, p62 serves as a substrate to be degraded in the autophagy-lysosome system via binding to ubiquitinated proteins. Accordingly, it is often used as a marker for autophagy degradation. However, there is a debate regarding autophagy biomarkers and their functions in IDD. Ye et al. reported significantly increased Beclin-1 and LC3-II/LC3-I protein expressions in IDD rats versus normal controls, supporting autophagy-induced IDD progression [42]. But most *in vitro* and *in vivo* experiments demonstrated a positive role of autophagy in IDD [43–45]. Liao et al. found that lipopolysaccharide-induced pyroptosis of human NP cells was relieved via autophagy-based unconventional secretory pathway stimulated by the autophagy inducer rapamycin compared to aggravated pyroptosis in cells treated with 3-MA [43]. Li et al. showed that autophagy activation decreased the expression of absent in melanoma 2 (AIM2) inflammasome against cellular DNA damage in rat NP cells via the autophagy-lysosome pathway [44]. Therefore, multiple autophagic signalings are involved in the pathogenesis of IDD. Blockade of autophagic flux impairs autophagosomal function and disrupts homeostasis of NP cell, thus initiating cell apoptosis [45]. The restoration of autophagy or autophagic flux for NP cell rescue or preventing IDD progression has been frequently demonstrated [24]. Therefore, protection against IDD can be autophagy-dependent.

Naringin is a natural flavonoid that has been confirmed to both stimulate and inhibit autophagy activity in many types of cells [46–48]. In this study, naringin induced autophagy increment and restored autophagic flux in human NP cells. Blocking autophagic flux by 3-MA sparked cell apoptosis and ECM component degradation in NP cells. All these support autophagy-mediated protection of naringin against NP cell death and IDD progression.

AMPK is a key energy sensor ubiquitous in eukaryotes and initiates autophagy to maintain energy homeostasis. During oxidative stress induced by ischemia and hypoxia, insufficient ATP supply, and the resultant high AMP content stimulate AMPK phosphorylation [49, 50], which activates numerous downstream substrates to inhibit ATP consumption and boost ATP production to maintain energy homeostasis. It also increases intracellular NAD^+^ levels to activate NAD^+^-dependent SIRT1 [51]. IVD pressure and nutrition are closely associated with AMPK/SIRT1-mediated restoration of autophagy in NP cells. In our study, naringin stimulated p-AMPK and SIRT1 expressions alongside elevated LC3 and Beclin-1 and lowered p62, which were offset, together with impaired autophagic flux, by the AMPK inhibitor EX-527 or SIRT1 silencing. So naringin protects against IDD through autophagy restoration via activation of the AMPK/SIRT1 signaling.

Some limitations are apparent. A single dose of naringin was insufficient for studying its efficacy. Multiple doses of naringin tested at several time points are required in our future studies. The roles of the autophagic AMPK/SIRT1 signaling were merely validated in vitro. Animal experiments for stronger evidence will be undertaken in the future publications in a timely manner. Furthermore, this study primarily focused on whether naringin's protection against IDD was autophagy-dependent and relevant mechanisms, rather than impaired biochemical homeostasis induced by proinflammatory cytokines and the potential mechanisms for the effect of naringin on rescuing it. However, the latter also deserves thorough exploration for a better understanding of this disease.

## 5. Conclusion

Naringin boosts autophagic flux through SIRT1 upregulation via AMPK activation, thus protecting NP cells against inflammatory response, oxidative stress, and impaired cellular homeostasis. Naringin seems a promising inducer for restoration of autophagic flux for IDD.

## Data Availability

The data that support the findings of this study are available from the corresponding author upon reasonable request.

## Abbreviations

IVD: Intervertebral disc
NP: Nucleus pulposus
ECM: Extracellular matrix
IDD: Intervertebral disc degeneration
LBP: Low back pain
TNF-*α*: Tumor necrosis factor-*α*
ROS: Reactive oxygen species
AMPK: AMP-activated protein kinase
SIRT1: Silent information regulator-1
3-MA: 3-Methyladenine
MMP-3: Matrix metalloproteinase-3
ADAMTS-4: A disintegrin and metalloproteinase with thrombospondin motifs-4
COX-2: Cyclooxygenase-2
SOD: Superoxide dismutase
AIM2: Absent in melanoma 2.
